# Multifunctional effects of *Lactobacillus sakei* HEM 224 on the gastrointestinal tract and airway inflammation

**DOI:** 10.1038/s41598-023-45043-0

**Published:** 2023-10-20

**Authors:** Hye-Shin Kim, Hanna Oh, Bobae Kim, Yosep Ji, Wilhelm H. Holzapfel, Hyeji Kang, Karina Arellano-Ayala

**Affiliations:** 1 Department of Advanced Convergence, Handong Global University, 558, Handong-ro, Pohang, Gyeongbuk 37554 Republic of Korea; 2 HEM Pharma Inc., Pohang, Gyeongbuk 37554 Republic of Korea; 3 Global Green Research and Development Institute, Handong Global University, Pohang, Gyeongbuk 37554 Republic of Korea; 4 Research Institute of Molecular Pathology (IMP), Vienna BioCenter (VBC), Campus-Vienna-Biocenter 1, Vienna, 1030 Austria

**Keywords:** Applied microbiology, Health care

## Abstract

Mucosal tissues serve as the first defense line and their commensal microbiota play a role in sustaining of host health. This study aimed to isolate and evaluate a putative probiotic strain on various mucosal regions. *Lactobacillus sakei* HEM 224 was isolated from traditional Korean kimchi and identified. In the safety assessment *L. sakei* HEM 224 showed negative results for hemolysis, biogenic amine production and transferable antibiotic resistance. The probiotic potential of strain HEM 224 in diverse mucosal areas was shown in two different models, viz. a murine model with colitis induced by dextran sulfate sodium (DSS) and an allergic airway inflammation model induced by ovalbumin (OVA). In the colitis model, oral administration of *L. sakei* HEM 224 improved colitis physiology with immunomodulation, enhancing barrier components and gut microbiota alteration. In the allergic airway inflammation model, the intranasal administration of the strain decreased type 2 inflammation and enhanced epithelial barrier integrity from the airways. These results demonstrate that *L. sakei* HEM 224 can ameliorate inflammatory conditions in both the gastrointestinal and respiratory tracts through the reinforcement of the epithelial barrier and immunomodulation.

## Introduction

Our body is composed of mucosal organs and specialized goblet cells and mucous cells are aligned in epithelia integrated by intercellular epithelial junctions. Their apical surface is covered by the glycocalyx matrix basically separating our body from the environment and trapping the influx of noxious stimuli^1^. Innate immune cells, antibodies and antimicrobial molecules are contained in the mucosal layer, and they counteract against invaders initiating adaptive immunity^2^. Moreover, each mucosal organ harbors a distinct microbiota and they actively interact with the barrier components to maturate host systems^3^. Gut and lungs are representative mucosal organs sharing structural and functional similarities. A proper mucus production is essential to sustain a steady-state of epithelial integrity and microbial homeostasis for both organs^4^. Therefore, the association between gut and lung (gut–lung axis) has been interesting and studied extensively in recent times.

Concerning pathogenesis of inflammatory diseases in the gut and lungs, both display a disrupted homeostasis resulting in mucosal inflammation, loss of barrier integrity, and dysbiosis^5,6^. Especially, the loss of epithelial integrity can trigger the exacerbation of diseases due to the influx of allergens, excess inflammatory cells and mediators, leading to a mucosal barrier damage. Thereby, increased intestinal permeability is one of representative features in inflammatory bowel diseases (IBD)^7^. In case of airway inflammation, inhaled harmful factors can disrupt barrier integrity connecting to the allergen sensitization. The loss and dysfunction of epithelial tight junctions as has been observed in asthmatic patients^8^.

Based on that, the use of probiotics has been proposed as one of the novel future strategies. Probiotics are defined as “living microorganisms, which when administered in adequate amounts, confer a health benefit on the host”^9^. Most probiotic strains belong to the lactic acid bacteria (LAB), especially to the genera *Lactobacillus* and *Bifidobacterium*, and they are often isolated from fermented foods^10^. Strains of *Lactobacillus sakei* have been used in the food industry as natural preservatives and starter cultures^11^. Recently, the aspects of immunomodulators of *L. sakei* have been highlighted and ameliorative effects on chronic inflammatory diseases in mucosal sites have been reported^12,13^. Likewise, several *L. sakei* strains showed potential as putative probiotics through immune system regulation, microbiota modulation and barrier reinforcement. Recently, several researchers have attempted to expand the application of *L. sakei* strains to distal parts such as pulmonary system, however, more research on *L. sakei* strain is still required. Based on the previous studies, this study aimed to investigate a novel and safe *L. sakei* strain from fermented food and to confirm its probiotic potential in in vitro and in vivo models, by specially targeting the gut and lung mucosal environments.

## Materials and methods

### Isolation

Korean fermented kimchi (pH 4.48) samples were homogenized in 1 × PBS (Lonza, Switzerland) followed by serial dilution, plating on De Man, Rogosa and Sharpe (MRS) (Becton, Dickinson and Company (BD), USA) and incubation under anaerobic conditions at 37 °C for 24 h. Colonies were selected for isolation and purification. First, a potassium hydroxide (KOH) test was conducted. A drop of 3% KOH solution was placed on a slide, and colonies from the pure culture were selected and mixed with the KOH solution. The resulting bacterial suspension did not exhibit a viscous characteristic, suggesting that the isolated strain is gram-positive. Subsequently, the picked colonies were exposed to a drop of hydrogen peroxide solution. The absence of bubbles upon reaction confirmed that the isolated strain is catalase negative.

The 16S rRNA sequencing was carried out by Solgent Inc (Daejeon, Republic of Korea). Bacterial DNA was isolated using the boiling method with Chelex beads (Bio-Rad, CA, USA). The amplification of the 16S rRNA gene was achieved through PCR, utilizing Solg™ EF-Taq DNA polymerase (Solgent Inc). Universal primers, 27F (AGA GTT TGA TCC TGG CTC AG), and 1492R (GGT TAC CTT GTT ACG ACT T) were employed. The PCR reactions were conducted in a Verti R TM 96-well Thermal Cycler (Thermo Fischer Scientific, MA, USA) with the following cycling conditions: 1 cycle of 95 °C for 15 min (initial denaturation); 30 cycles of 95 °C for 20 s (denaturation); 30 cycles of 50 °C for 40 s (annealing); 30 cycles of 72 °C for 1 min 30 s (extension); followed by 1 cycle of 72 °C for 5 min (final extension). Sequencing was executed using an ABI PRISM 3730XL DNA analyzer from Applied Biosystems, USA. The homologous sequences obtained from the results were identified using The Basic Local Alignment Search Tool (BLAST) in GenBank.

### Bacteria

*L. sakei* HEM 224 and *L. rhamnosus* GG were grown on MRS agar and in broth (BD) at 37 °C for 16 h. As pathogenic controls for hemolysis and the biogenic amine production assay, *Escherichia coli* ATCC 25922 and *Bacillus cereus* ATCC 27348 were used. Both strains were cultured in Brain Heart Infusion (BHI) (BD) media at 37 °C for 18 h with agitation.

### Whole genome sequencing

The complete genome sequence of *L. sakei* HEM 224 was generated using the PacBio RS platform with single molecule real-time (SMRT) sequencing at Theragenetex Inc. (Seoul, Republic of Korea) as commercial service. Briefly, *L. sakei* HEM 224 was cultured on MRS agar, as previously described. Bacterial DNA was extracted from a fresh pellet using the Wizard^®^ Genomic DNA Purification Kit (Promega, Thermo Fischer Scientific). Subsequently, libraries were prepared for 151 bp paired-end sequencing using the TruSeq Nano DNA Sample Prep kit (Illumina, CA, USA), and sequencing was conducted as paired-end (2 × 151 bp) with the Illumina NovaSeq platform. Following SMRT library preparation and sequencing, a draft genome was assembled using CANU v.1.7. Annotations were performed by merging the results obtained from the Rapid Annotations using Subsystems Technology (RAST) server^14^.

### Hemolysis

The bacterial cultures grown at 37 °C for 16 h were streaked onto blood agar plates containing 5% of sheep blood (Hanil Komed, Republic of Korea), followed by incubation at 37 °C for 24 h to 48 h. The hemolysin production was determined by observation of the clear zone formation.

### Biogenic amine formation

*L. sakei* HEM 224 was cultured in a special medium with some modifications at 37 °C for 48 h^15^. Briefly, 1% of each precursor amino acids [l-tyrosine (Samchun Chemicals, Republic of Korea), l-histidine (Daejung Chemicals, Republic of Korea), l-ornithine (Sigma-Aldrich), and l-lysine (Samchun Chemicals)] was included in each decarboxylase medium to determine the production of tyramine, histamine, putrescine and cadaverine. The production ability was identified by color change of the pH indicator bromocresol purple in the medium.

### Antibiotic susceptibility

The agar diffusion method was used to determine the antibiotic susceptibility of the strains suggested by the Clinical and Laboratory Standard Institute (CLSI; http://www.clsi.org) and/or EFSA. The method previously described was introduced with some modifications^16^. Two-fold serial dilutions of antibiotic solutions were prepared and suspended in MRS and Iso-Sensitest mixture agar (Oxoid, UK). The bacterial suspension was adjusted to desired concentration (1 × 10^7^ CFU/mL) and 10 μL (1 × 10^5^ CFU/mL) was inoculated onto the plates using a multipin-inoculator. The plates were incubated at 37 °C for 24 h under anaerobic conditions. Antibiotic resistance was determined by a higher concentration of a specific antimicrobial than the cut-off value according to the European Food Safety Authority^17^. The presence of acquired antimicrobial resistance genes in whole-genome data were identified by using the ResFinder program (https://cge.cbs.dtu.dk/services/ResFinder/)^18,19^.

### Simulated stomach duodenum passage (SSDP)

The assay was performed as previously described^20^. Strains for SSDP were inoculated at a level of 1% into MRS broth at pH 3.0 and incubated at 37 °C. After 1 h of incubation, duodenum juice (NaHCO3: 6.4 g/L, KCl: 0.239 g/L and NaCl: 1.28 g/L) and bile acid (10% oxgall; BD) were added promptly and the medium was incubated at 37 °C for two additional hours. The viable colonies were counted after 0 h, 1 h and 3 h.

### Cell adhesion

Caco-2, the human colorectal epithelial cell-line (Korean Cell Line Bank, Republic of Korea), were grown in Minimum Essential Medium Eagle (MEM) (Corning^®^, USA) supplemented 10% fetal bovine serum (Gibco™, USA), 1% antibiotics (antibiotic–antimycotic, Gibco™) and 1% non-essential amino acids solution (Gibco™) at 36.5 °C and 5% CO_2_ environment. Cells (1 × 10^5^ cells/well) were seeded in 12 well plate and maintained until confluency. Bacteria cell adhesion assay was conducted according to the previous protocol with some modification^21^. Briefly, *L. sakei* HEM 224 bacterial cells (1 × 10^7^ cells/well) were harvested and washed after 16 h of incubation following resuspension in MEM. Caco-2 cells were washed to remove antibiotics and the bacterial suspension was treated at a ratio of 100:1 (bacterial cells:Caco-2 cells) for 2 h. After co-incubation, the number of attached bacteria was counted on MRS agar plates after 48 h of incubation at 37 °C.

### In vivo DSS-induced colitis model

Seven-week-old female C57BL/6J mice were purchased from Central Laboratory of Animal Inc. (Seoul, Republic of Korea). Mice were adapted for 1 week and housed under controlled environmental condition (23–25 °C, 40–60% of humidity and 12 h light/dark cycle) with chow diet and water ad libitum. To induce colitis in mice, 4% DSS salt (MP Biomedicals, Santa Ana, USA) in drinking water was administered for a period of 6 days, followed by a washout phase using sterile water for 2 days. *L. sakei* HEM 224 (1 × 10^9^ CFU/200 μL/mouse/day) was orally administered from 2 weeks before DSS treatment until the day before sacrifice. Mixture of ketamine hydrochloride (Yuhan, Republic of Korea) and xylazine (Rompun) (Korea branch of Elanco Co., Republic of Korea) was used for anesthetizing and sacrificing by intraperitoneal route. In detail, the composition of ketamine/xylazine is following: 3.2 mL of ketamine hydrochloride, 1.4 mL of xylazine and 5.4 mL of PBS. The control group received PBS only. All experimental protocols were approved by Institutional Animal Care and Use Committee (IACUC) of Handong Global University (approval number HGUIACUC 20191008-021) and all procedure were carried out in compliance with the ARRIVE guideline and the Guidelines for Animal Care and Use at Handong Global University.

### In vivo OVA-induced allergic airway inflammation model

Six-week-old female BALB/c mice were housed under specific pathogen-free conditions. The allergic airway inflammation was induced to mice based on the previous protocol with some modifications^22^. Mice were sensitized with 50 μg of OVA (Sigma-Aldrich, USA) with 2 mg of aluminum hydroxide (alum) (Sigma-Aldrich) on days 0 and 7 via the intraperitoneal route. After the last sensitization, the mice were intranasally challenged with 10 μg of OVA on days 14, 17, 21, 24, 28 and 31. On day 32, mice were sacrificed and serum, bronchoalveolar lavage fluid (BALF) and lung tissue were collected. *L. sakei* HEM 224 (1 × 10^7^ CFU/mouse) was administered via the intranasal route after the first allergen challenge. The administration was performed after anesthesia for five consecutive days per week for 3 weeks before sacrifice. Mixture of ketamine hydrochloride (Yuhan) and xylazine (Rompun) (Korea branch of Elanco Co.) was used for anesthetizing and sacrificing. All experimental protocols were approved by Institutional Animal Care and Use Committee (IACUC) of Handong Global University (approval number HGUIACUC 20190328-013) and all procedure were carried out in compliance with the ARRIVE guideline and the Guidelines for Animal Care and Use at Handong Global University.

### Histology

The distal colon (~ 2 cm) and left superior lobes of the mouse lung were fixed in 10% formaldehyde for histological analysis including hematoxylin and eosin (H&E) and periodic acid-Schiff (PAS) staining at KP&T Inc. (Cheongju, Republic of Korea) as commercial service. In brief, the fixed tissues were embedded in paraffin, and 3-μm-thick slices were prepared. After deparaffinization and dehydration, H&E or PAS staining was carried out.

### ELISA

The protein levels of IL-4 and IL-5 in BALF were measured by using ELISA MAX™ Deluxe Set Mouse IL-4 and IL-5 (BioLegend, USA) according to the manufacturer’s protocol. For serum IgE analysis, the obtained serum was stored at − 80 °C and measured using ELISA MAX™ Deluxe Set Mouse IgE (BioLegend) according to the manufacturer’s instructions.

### mRNA extraction and quantitative real-time PCR analysis

The mRNA from each tissue was extracted with TRIzol (Thermo Fischer) according to the manufacturer’s protocol. The extracted mRNA was reverse transcribed into complementary DNA with GoScript™ Reverse Transcription kit (Promega, Thermo Fischer Scientific). qRT-PCR was performed by TB Green™ Premix Ex Taq kit (Takara Bio, Japan) using Step-One Plus real-time PCR system (Applied Biosystems, USA), with gene-specific primers for acidic ribosomal phosphoprotein p0 (ARBP), β-actin, C–C motif chemokine ligand (CCL) 22, CCL24, claudin (CLDN) 3, CLDN 4, CLDN 7, CLDN 13, CLDN 18, interleukin (IL) 1β, IL-6, IL-10, IL-13, IL-17, IL 22, mucin (MUC) 4, MUC5AC, occludin (OCLDN), tumor necrosis factor α (TNF-α), zonula occludens-1 (ZO-1). Information on the primers used is listed in Table S1. The amplification process followed these reaction conditions: an initial denaturation step of 2 min at 95 °C, 45 cycles of 15 s at 95 °C and 1 min at 60 °C. After the amplification, a melting curve analysis was carried out, involving the following steps: 15 s at 95 °C, 60 s at 60 °C, and heating up (0.3 °C/s). Data were quantified by the comparative threshold cycle method. The colon data was normalized by ARBP and lung data with β-actin gene expression.

### Short chain fatty acid (SCFA) analysis

SCFA analysis was performed using Headspace sampler-gas chromatography-Flame ionization detector (HSS-GC-FID) analysis. To provide a brief overview, all SCFAs were extracted from 0.1 g of mice fecal samples in 1 mL of distilled H_2_O. These samples were vortexed and then centrifuged at 13,000 rpm for 3 min at room temperature. The resulting supernatant was transferred to a vial containing a GC buffer solution, and 2-ethylbutyric acid was added as an internal standard^23^. The samples were analyzed using an Agilent 7890B GC system equipped with a 7697A headspace sampler and FID (Agilent Technologies, USA). Data acquisition and processing were performed using ChemStation software from Agilent Technologies.

### DNA extraction and next generation sequencing (NGS) analysis

DNA extraction and NGS analysis were conducted by HEM Pharma Inc. Briefly, bacterial DNA in feces was extracted and the fecal sample was homogenized by bead-beating. After isolating, cleaning, and eluting procedures based on the manufacturer’s protocols, the variable V3–V4 regions of the 16S ribosomal RNA gene was sequenced by Illumina Miseq System (Illumina). Sample DNAs were cleaned and the Index PCR was carried out. The concentration of libraries was verified and sequenced using Illumina Miseq system. Reads were sorted using the unique barcodes for each PCR product. The barcode, linker and primer sequences were then eliminated from the original sequencing reads for the next analysis step.

### Bioinformatic analysis

Single end FASTQ files were imported into the Quantitative Insights into Microbial Ecology 2 (QIIME2, ver. 2021.2) via the FASTQ manifest protocol^24,25^. Demultiplexed files were denoised with DADA2^26^. All amplicon sequence variants were aligned with MAFFT plugin and a phylogeny analysis was performed with Fasttree2 based on the Greengenes database^27,28^. For all analyses, all sampling depths reached to 19,400 reads. Alpha‐diversity metrics (Shannon index^29^, Simpson index^30^, Chao1 index^31^, Evenness^32^ and Faith’s Phylogenetic Diversity^33^), beta diversity metrics (weighted UniFrac^34^, unweighted UniFrac^35^), and principal coordinate analysis (PCoA) were performed to evaluate the microbial distances between groups.

### Statistical analysis

Data were analysed by GraphPad Prism software 6.0 and 9.3.1 (GraphPad Software, USA) and expressed as the mean ± SD. Statistical significance was calculated by one-way ANOVA with Fisher’s LSD test.

## Results

### Isolation and identification of *L. sakei* HEM 224

**Figure 1:**
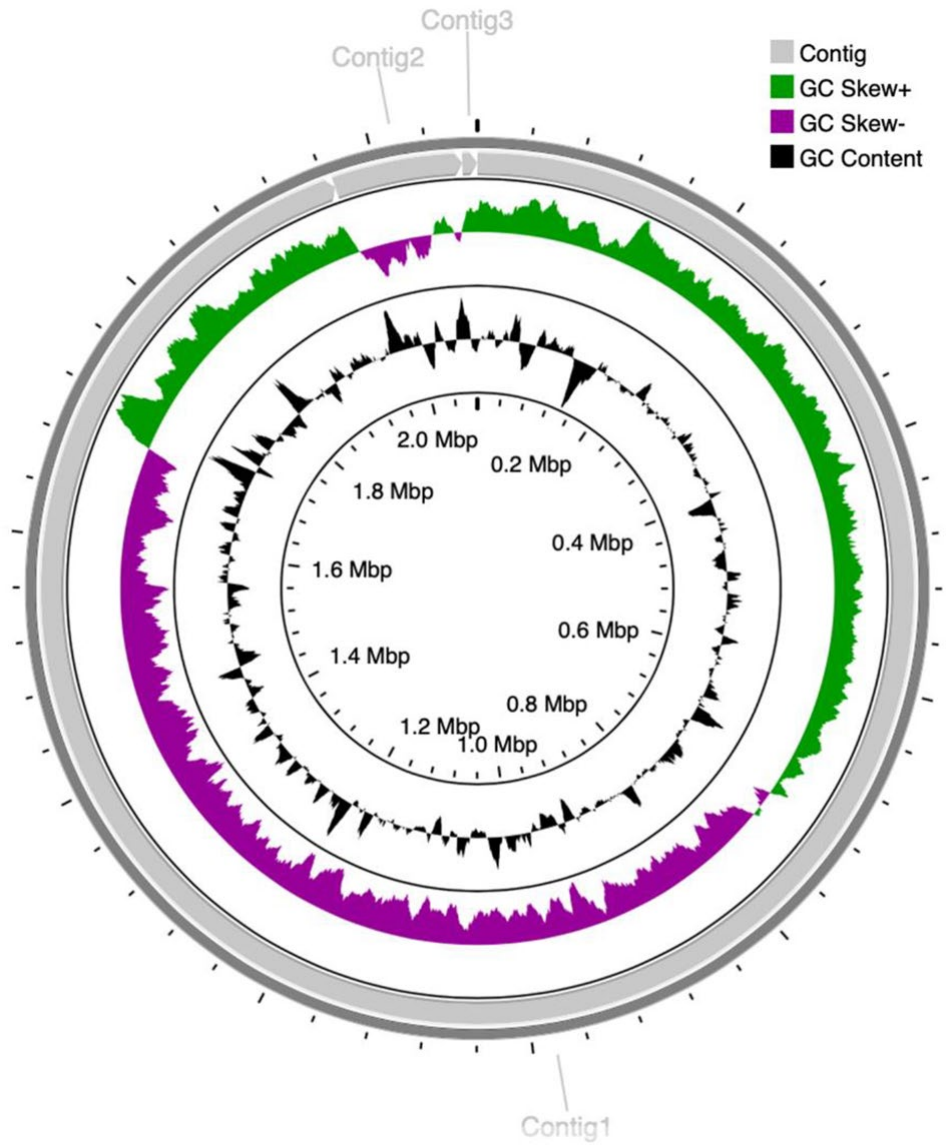
The circular genome map of *L. sakei* HEM 224. The first circle (starting with the inner circle) represents the genome size. The second circle indicates the GC content. The third circle depicts the GC skew (G + C/G − C). The fourth circle shows the number and location of contigs.

Lactic acid bacterial strains were isolated from a vegetable-based kimchi ingredient and predominantly identified as *L. sakei* by comparative 16S rRNA sequencing. Among the identified isolates, a strain with negative catalase reaction, *L. sakei* HEM 224, was selected for further investigation (data not shown). To obtain entire genome information of *L. sakei* strain HEM 224, whole genome sequencing was performed. The genome of the strain was composed of a circular DNA chromosome with a total size of 2,079,011 bp with a G + C content of 41.2 mol%. Three contigs were found with the varying in range from 11,771 to 1,966,385 bp (Fig. 1).

### Safety evaluation of *L. sakei* HEM 224

To determine the safety of the isolated strain, hemolytic activity, biogenic amine production and antibiotic susceptibility were evaluated. *B. cereus* ATCC 27348 served as a positive control in the hemolysis test and *L. sakei* HEM 224 showed no hemolysis reaction. In the biogenic amine production assay, strain HEM 224 was confirmed be negative in presence of the precursor amino acids compared to a positive control, *E. coli* ATCC 25922 (Table S2). The strain *L. sakei* HEM 224 was also examined for antibiotic susceptibility and all the minimum inhibitory concentration (MIC) values of the target strain were below or similar to the compared EFSA cut-off values, indicating its susceptibility to all tested antibiotics (Table S3). Moreover, the presence of acquired antibiotic resistance genes was assessed through the ResFinder program and *L. sakei* HEM 224 was confirmed to negative for any resistance genes of various classes of antibiotics (Table S4). These data suggested *L. sakei* HEM 224 to be safe for human consumption and thereby it qualified for further evaluation as a probiotic candidate.

### Adaptability of *L. sakei* HEM 224 to GIT

In vitro SSDP assay was performed to determine the potential viability of *L. sakei* HEM 224 under conditions of the upper GIT. The strain was exposed to a simulated stomach- and duodenum-like environment in sequence and counted at each timepoint. *L. sakei* HEM 224 showed a higher survival rate after the stomach- and duodenum-simulated environment pass than that of LGG (Table S5). Additionally, the adhesion ability of strain HEM 224 was also investigated using Caco-2 cells. After incubation, its adhesion rate of was similar to that of LGG (Table S6). These data suggest that *L. sakei* HEM 224 has a comparable or superior adaptability to upper GIT conditions as the probiotic control.

### *L. sakei* HEM 224 confers protective effect against DSS-induced colitis

**Figure 2:**
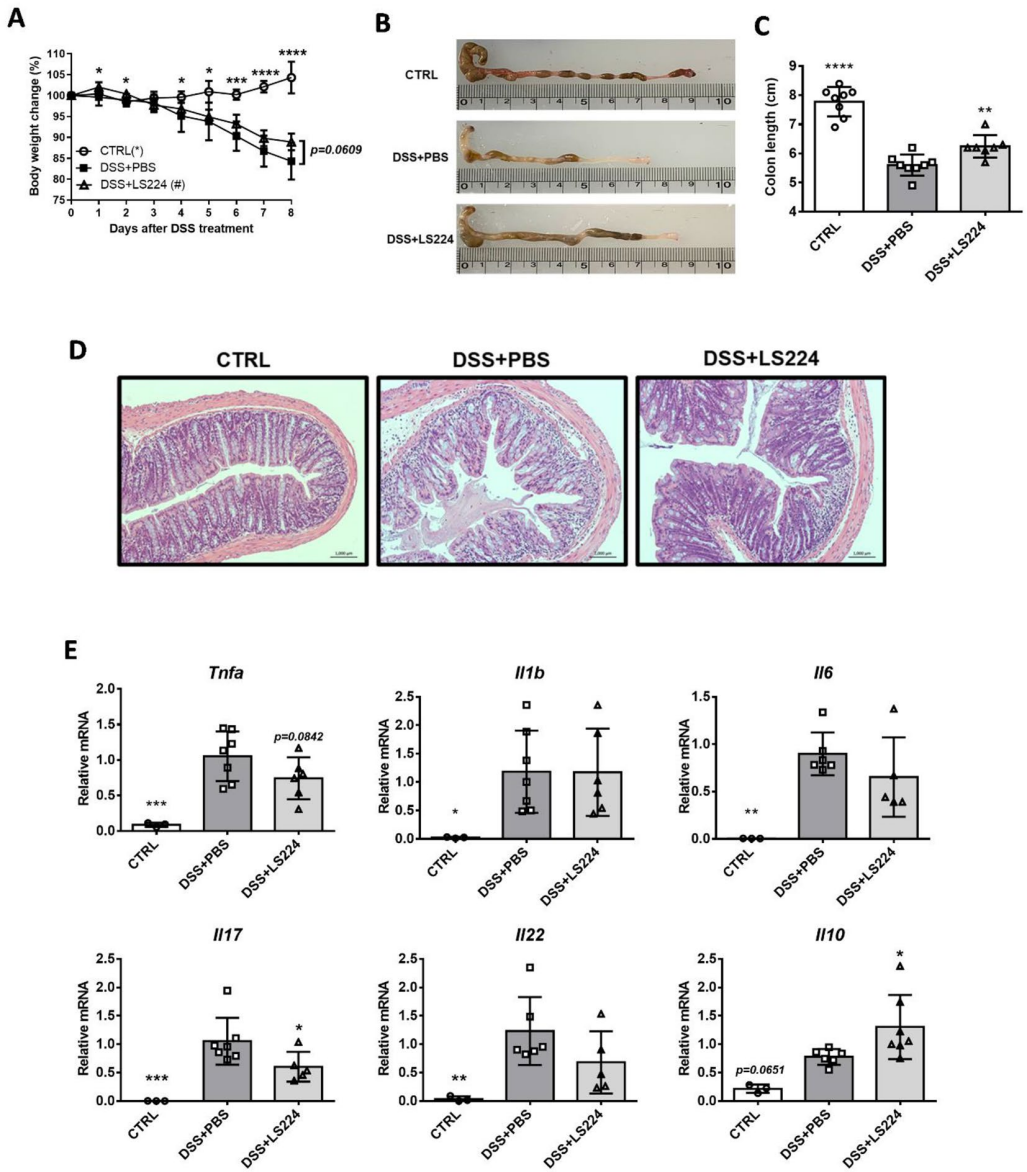
Effects of oral administration of *L. sakei* HEM 224 in mice with DSS-induced colitis. (**A**) Body weight, (**B**) the representative gross appearance of the colon measurement, (**C**) colon length, (**D**) histopathology of distal colon by hematoxylin and eosin (H&E) staining, (**E**) relative mRNA expression of cytokines in murine colon tissue measured by qRT-PCR. *CTRL* vehicle-treated control, *DSS + PBS* DSS + vehicle-treated group, *DSS + LS224* DSS + *L. sakei* HEM 224 treated group. Data show the mean ± SD (n = 6–8 mice per group) and the difference compared to the DSS + PBS group was analyzed by one-way ANOVA with Fisher’s LSD test. *p < 0.05, **p < 0.01, ***p < 0.001, ****p < 0.0001.

To evaluate the functionality of *L. sakei* HEM 224 in an in vivo system, its alleviative potential was examined against a DSS-induced colitis model. The oral consumption of *L. sakei* HEM 224 ameliorated body weight loss and recovered colon length significantly (Fig. 2A–C). Histological specimens showed that *L. sakei* HEM 224 treatment improved a deteriorated structure of epithelial barrier (Fig. 2D). In the colon, *L. sakei* HEM 224 downregulated the mRNA expression of major pro-inflammatory cytokines (Fig. 2E).

### *L. sakei* HEM 224 improves gut epithelial barrier by upregulation of TJPs in the gut

**Figure 3:**
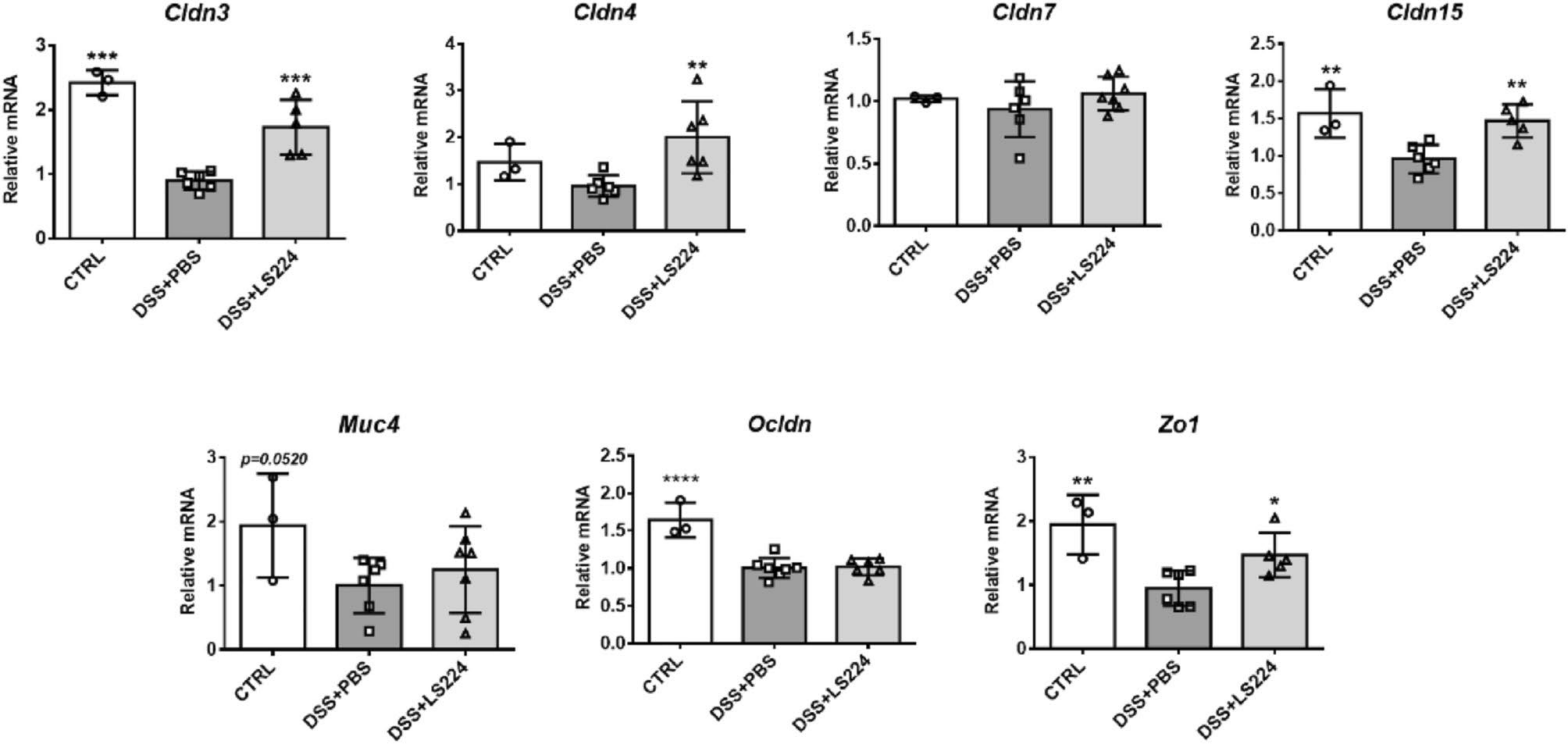
Effects of *L. sakei* HEM 224 on tight junction protein and mucin expression in mice with DSS-induced colitis. The relative expression of genes regarding tight junction proteins in murine colon tissue was measured by qRT-PCR. *CTRL* vehicle-treated control, *DSS + PBS* DSS + vehicle-treated group, *DSS + LS224* DSS + *L. sakei* HEM 224 treated group. Data show the mean ± SD (n = 6–8 mice per group) and the difference compared to the DSS + PBS group was analyzed by one-way ANOVA with Fisher’s LSD test. *p < 0.05, **p < 0.01, ***p < 0.001, ****p < 0.0001.

Moreover, the TJP expression levels in the colon were measured and the strain significantly upregulated main TJP markers (Fig. 3). In case of mucin, the gene expression of MUC4 showed an increasing tendency only. Collectively, this strain may reinforce the epithelial barrier integrity with the upregulation of TJPs, thereby resulting in a protective effect against colitis.

### *L. sakei* HEM 224 modulates gut microbial metabolites and gut microbiota

**Figure 4:**
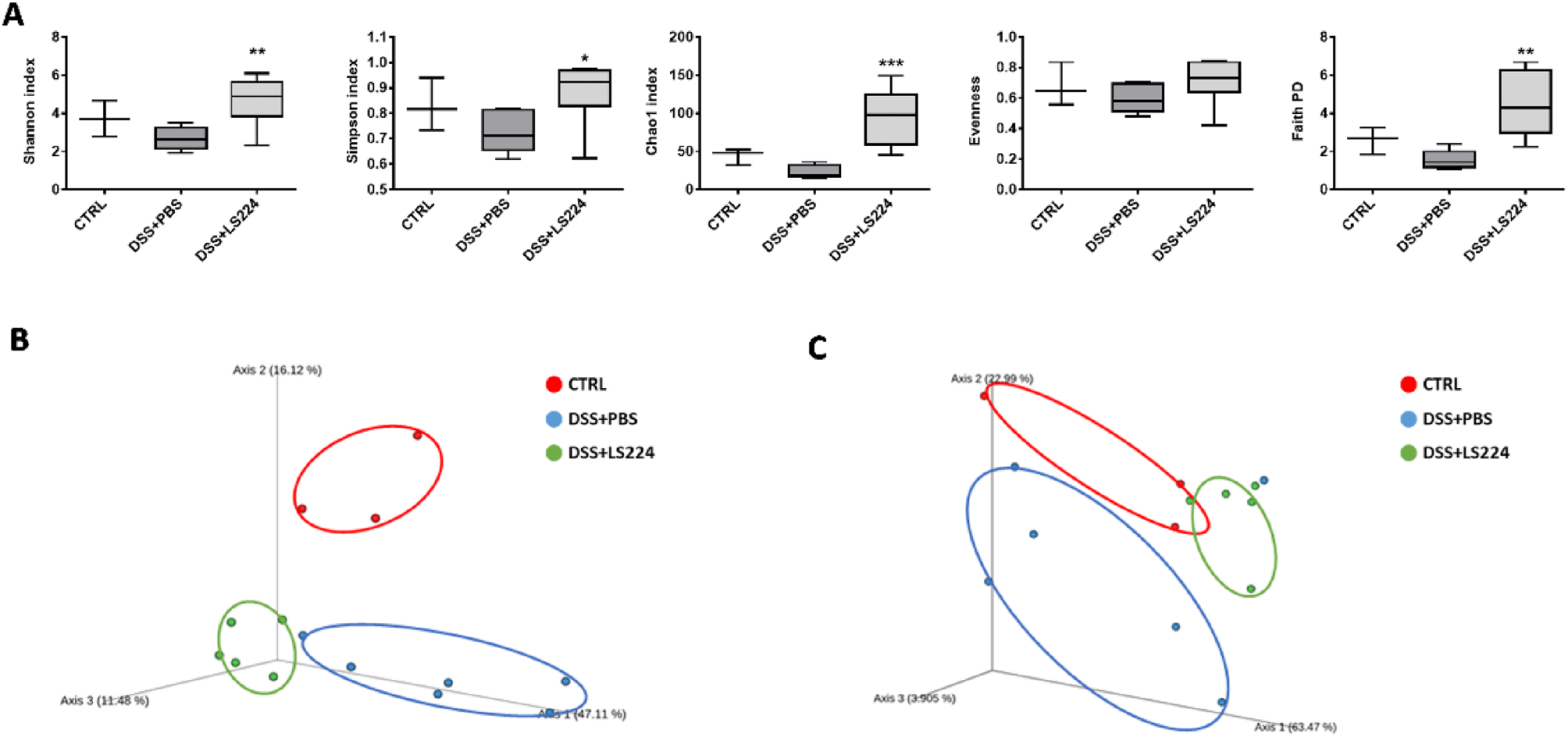
Effects of oral administration of *L. sakei* HEM 224 on microbiota modulation in mice with DSS-induced colitis. (**A**) Bar plots of alpha diversity indexes (from left; Shannon, Simpson, Chao1, evenness and Faith PD), (**B**) principal coordinates analysis (PCoA) derived from unweighted and (**C**) weighted UniFrac. *CTRL* vehicle-treated control, *DSS + PBS* DSS + vehicle-treated group, *DSS + LS224* DSS + *L. sakei* HEM 224 treated group. Alpha diversity data show the mean ± SD (n = 3–6 mice per group) and the difference compared to the DSS + PBS group was analyzed by one-way ANOVA with Fisher’s LSD test. *p < 0.05, **p < 0.01, ***p < 0.001.

In the SCFA profiles of the three experimental groups, butyrate production level was significantly increased in the *L. sakei* HEM 224-treated group suggesting the possible modulation of the gut microbiota (Table S7). Subsequently, *L. sakei* HEM 224 ingestion resulted in increased values in every alpha diversity index (Fig. 4A). The microbial community structure was analyzed based on beta diversity including unweighted and weighted UniFrac distances and represented by principal coordinates analysis (PCoA) plot. Each group presented a distinct clustering and especially the *L. sakei* HEM 224 treatment group showed a clear difference compared to the DSS + PBS group (Fig. 4B). In the taxonomical analysis the *L. sakei* HEM 224 group augmented the abundance of *Lactobacillus* and *Clostridium* species groups (data not shown). The microbial data suggest that the *L. sakei* HEM 224 induced the modulation of the gut microbiota.

### *L. sakei* HEM 224 ameliorates allergic airway inflammation

**Figure 5:**
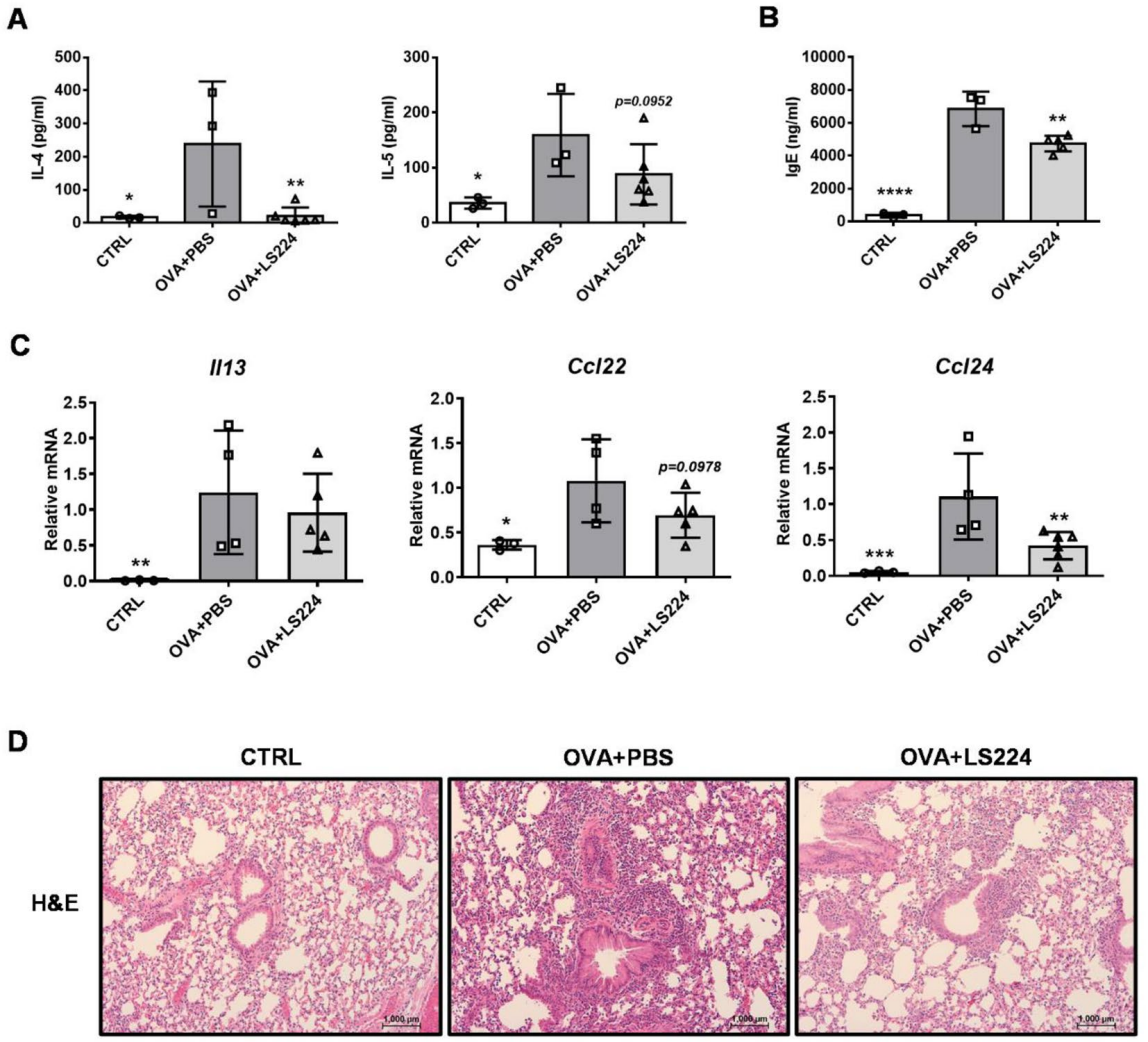
Effects of intranasal administration of *L. sakei* HEM 224 on type 2 inflammation in an OVA-induced allergic airway inflammation murine model. (**A**) Th2 cytokines in BALF, (**B**) IgE in serum, (**C**) mRNA expression of type 2 cytokines and chemokines and (**D**) lung histology (H&E). *CTRL* vehicle-treated control group, *OVA + PBS* OVA + vehicle-treated group, *OVA + LS224* OVA + *L. sakei* HEM 224 treated group. Data show the mean ± SD (n = 3–6 mice per group) and the difference compared to the OVA + PBS group was analyzed by one-way ANOVA with Fisher’s LSD test. *p < 0.05, **p < 0.01, ***p < 0.001, ****p < 0.0001.

In order to assess the immunomodulatory effect of the strain in a local mucosal organ, an allergic airway inflammation model with induction by OVA was applied. Intranasal administration of *L. sakei* HEM 224 successfully decreased IL-4 and IL-5 in BALF and IgE in the serum (Fig. 5A,B). IL-13 decreased at a transcript level but with no significance. The mRNA expression of chemokines was also altered and especially, C–C motif chemokine ligand (CCL) 24 was significantly reduced (Fig. 5C). In the histological analysis, the infiltration of inflammatory cells was mitigated around the alveoli in the *L. sakei* HEM 224 treated group (Fig. 5D).

### *L. sakei* HEM 224 improves airway epithelial barrier by upregulation of TJPs and inhibition of mucus hyperproduction in the lungs

**Figure 6:**
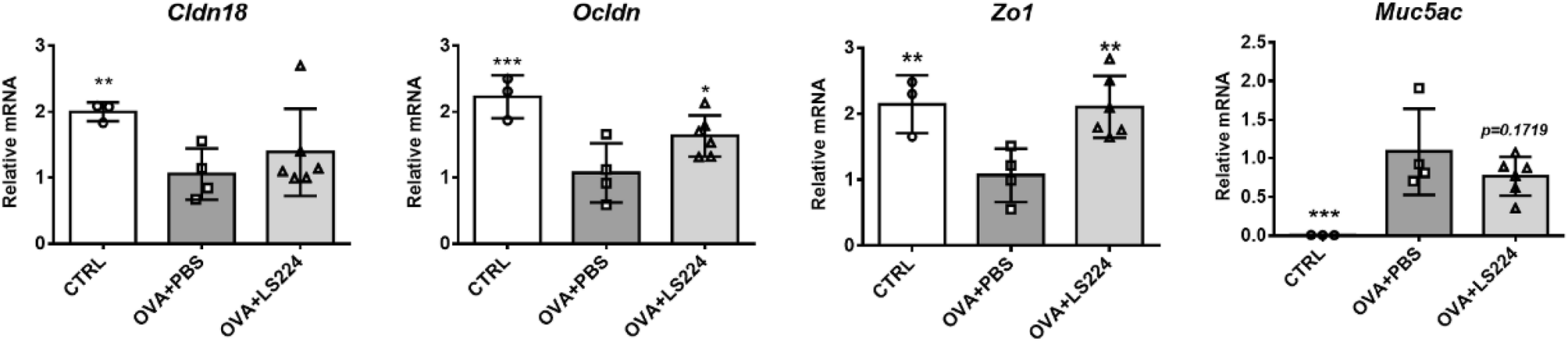
Effects of intranasal administration of *L. sakei* HEM 224 on the tight junction proteins and mucin expression in an OVA-indued allergic airway inflammation murine model. The relative mRNA expression of cytokines in murine lung tissue was measured by qRT-PCR. *CTRL* vehicle-treated control group, *OVA + PBS* OVA + vehicle-treated group, *OVA + LS224* OVA + *L. sakei* HEM 224 treated group. Data show the mean ± SD (n = 3–6 mice per group) and the difference compared to the OVA + PBS group was analyzed by one-way ANOVA with Fisher’s LSD test. *p < 0.05, **p < 0.01, ***p < 0.001.

**Figure 7:**
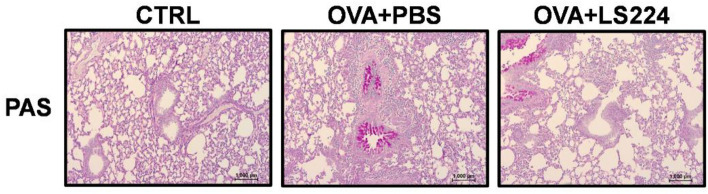
Effects of intranasal administration of *L. sakei* HEM 224 on mucus hypersecretion in an OVA-indued allergic airway inflammation murine model. Images of Periodic-Acid Schiff (PAS) staining (original magnification, × 100).

Next, the effects of intranasal treatment of *L. sakei* HEM 224 on airway epithelial barrier health were evaluated about expression of TJP genes and mucus hypersecretion. The mRNA expression levels of occludin and ZO-1 were significantly increased compared with the OVA + PBS group. Moreover, the MUC5AC mRNA expression levels showed a decreasing tendency (*p* = 0.1719) suggesting the improvement of the epithelial barrier integrity in the airways (Fig. 6). Intranasal treatment of *L. sakei* HEM 224 still showed the increased amount of mucus along with the alveoli surface (violet), but the intensity was alleviated compared to the OVA + PBS group (Fig. 7). These results demonstrate that the intranasal administration of *L. sakei* HEM 224 ameliorated allergic airway inflammation and improved airway epithelial barrier health, impacting TJP expression and mucus hypersecretion.

## Conclusion and discussion

Gut and lungs possess several similarities connecting to the overlapped characteristic regarding pathogenesis, therefore, the connection between two distinct organs has been highlighted. Inflammatory diseases in both sites accompany dysregulation not only of the immune response but also of the epithelial barrier and mucus production. It was reported that patients with IBD suffer from barrier dysfunction with the reduction of goblet cells and reduced thickness of the mucus layer, and of impaired TJPs leading to increased severity^36^. The relationship between epithelial permeability and inflammation is similar to that of the respiratory system. The loss or dysfunction in TJPs is linked to the pathogenesis of various airway disorders. A reduced gene expression of TJPs such as ZO-1 and occludin was observed as inflammatory phenotypes of an airway inflammation in vivo model^37,38^. Therefore, the well-tightened epithelial lining might be considered a key factor for protection in mucosal diseases.

Probiotics have been suggested as alternatives for treatment of various inflammatory diseases in mucosal sites due to their advantages; the modulation of the (gut) microbiota, the maturation of the immune system and reinforcement of the epithelial barrier^39^. Here, we suggested that the novel putative probiotic strain *L. sakei* HEM 224, assessed for its safety and functionality, to have beneficial potential for alleviating various mucosal diseases.

This study successfully isolated a novel *L. sakei* strain and acquired its genomic information through whole genome sequencing (Fig. 1). Although most LAB are considered as safe, safety assessment on a strain level is essential to determine any potential risk to consumers^40^. The biogenic amine formation assay aims to evaluate whether the tested strain can produce specific biogenic amines by decarboxylating of precursor amino acids in the test medium. Biogenic amines exist in low concentrations in nature and low consumption does not elicit harmful effects; however, elevated levels in the diet and the human body can be toxic leading to a health hazard, especially for sensitive persons^41^. *L. sakei* HEM 224, was identified to be negative to the tested biogenic amines (Table S2).

Antibiotic resistance may be a critical issue especially when related to the presence of acquired resistance genes, indicating the problematic potential of transferability^42^. *L. sakei* HEM 224 was susceptible to all tested antibiotics recommended by EFSA^17^ (Table S3). Furthermore, the presence of acquired resistance genes regarding several antibiotic classes was screened for using ResFinder program and no acquired resistance genes were detected (Table S4).

Regarding functionality evaluation, tolerance to the acidic environment of the stomach is a desirable requirement of putative probiotics to reach the intestine and ultimately elicit beneficial effects^43^. The adherence of a strain to the gut epithelium is closely associated with colonization and eventually the expression of beneficial functions^44^. Our strain showed notable survivability after passage of the gastric- and duodenum-like environments and a similar attachment ratio to intestinal epithelial cells, comparable to that of the representative commercial probiotic strain, LGG, in an in vitro study (Tables S5, S6).

The probiotic potential of *L. sakei* HEM 224 was further investigated in an in vivo mouse model with induced colitis and allergic airway inflammation. The DSS-induced colitis model has been reported to closely resemble human ulcerative colitis and has therefore been extensively used in IBD research^45^. In our experiments, DSS-treated mice showed a significant weight loss, colon shortening and a disrupted villi structure in histological analysis, however, *L. sakei* HEM 224 supported the recovery of physiology markers (Fig. 2A–D). The mRNA expression of pro-inflammatory cytokines was also suppressed and that of IL-10, the well-known anti-inflammatory regulatory cytokine, was increased (Fig. 2E). It was reported that a specific *L. sakei* strain can modulate the immune system by boosting the number of regulatory T cells (Treg) and IL-10 production^46^. However, our strain could not influence the increase IL-10 production in the colon (data not shown). Therefore, we expected epithelial barrier markers may be responsible for the mechanism(s). TJPs exist in the distal colon and decreased levels of them indicate the disruption of the gut barrier^47^. The loss of mucin production leads to the weakening of the intestinal barrier and to increased permeability, a typical feature of IBD^48^. In this study, *L. sakei* HEM 224 increased the mRNA expression level of both TJPs and mucin markers (Fig. 3). These data strongly suggest that *L. sakei* HEM 224 can reinforce the gut barrier integrity by upregulation of TJPs and mucin.

Another possible mechanism for alleviating colitis in mice is through the modulation of gut microbiota. It was reported that the application of probiotic strain could modulate gut microbiota with the increase in diversity and the ratio of Bacteroidetes and Firmicutes, and eventually attenuated the diseased conditions^49^. In this study, the gut microbiota diversity of the *L. sakei* HEM 224 treated group was increased and showed a clear clustering in beta diversity (Fig. 4). These data confirm that *L. sakei* HEM 224 oral administration induced the gut microbiota modulation. Taxonomic analysis did not show any significant difference at the phylum level, however, an increased abundance of *Lactobacillus* and *Clostridium* species was observed (data not shown), suggesting that our strain may modulate the gut microbiota and contribute to forming a favorable environment for LAB.

Bacterial metabolites have been suggested as possible factors for strengthening the epithelial barrier by upregulating tight junctions. Probiotics can induce the production of metabolites such as SCFA and stimulate mucin secretion and repair of tight junctions^50^. SCFAs are representative and effective metabolites that can directly or indirectly affect gut barrier integrity. Specifically, butyrate can induce the tight junction component gene contributing to the enhancement of the epithelial barrier function. Moreover, butyrate production is closely related to the gut microbiota modulation in the chronic inflammatory state^51^. In our study, the *L. sakei* HEM 224 treated mice showed an increased butyrate production more specifically than for any other SCFAs (Table S7). We assumed that butyrate may be involved in the upregulation of tight junction genes and gut microbiota modulation as well.

*L. sakei* has been applied not only to the gut but also other local mucosal organs, but the application to pulmonary system is sparse. The respiratory tract is the second-largest mucosal organ after the GIT and is endlessly challenged by numerous external factors. The innate immune system competes against inhaled antigens and both resident and infiltrated immune components sustain a proper immune tone to avoid unwanted airway inflammation^52^. However, the immune balance can be disrupted for various reasons including the genetic background and external influences and can lead to respiratory inflammatory conditions. In the inflamed condition, infiltrated immune cells worsen the response, impacting the epithelial barrier structurally and functionally^53^. Similar to IBD studies involving probiotics, beneficial microbes have been proposed as potential alternatives for treatment of airway inflammation. The administration of several *Lactobacillus* strains showed alleviative effects including immunomodulation and gut microbiota modulation against allergic airway inflammation model^54^.

Research on probiotics and airway inflammation predominantly focused on the oral administration of bacterial strains, while direct targeting of respiratory sites has become a recent focus when beneficial effects of direct contact have been observed^55^. In this study, *L. sakei* strain HEM 224 was administered intranasally to an OVA-induced allergic airway inflammation model. The results showed successful suppression of Th2-related allergic reaction markers and improvement in physiology was observed in the histological analysis (Fig. 5). Interestingly, when the strain was applied by the oral route, the strain could not modulate biased-Th2 reaction (data not shown). These results indicate that the treatment with *L. sakei* HEM 224 can impact the target site when it is in direct contact with the organ.

Another key point of this study is that *L. sakei* HEM 224 upregulated TJP genes expression in the lungs, similar to the colitis results. Chronic airway inflammation can cause airway epithelial damage together with the downregulation of specific TJPs^37^. *L. sakei* HEM 224 augmented the mRNA expression of claudin-18, occludin, and ZO-1 in the lungs (Fig. 6), suggesting the recovery effect of the strain on the impaired epithelial integrity. Unlike the colitis model, mucin hypersecretion is problematic in the allergic airway inflammation model. MUC5AC/*muc5ac*, the representative secretory mucin in the airway, has been reported as a marker for goblet cells on the airway surface and patients with asthma showed abnormal production^56^. In our airway inflammation model, intranasal treatment with *L. sakei* HEM 224 downregulated the mRNA expression of MUC5AC (Fig. 6) and confirmed the suppressed production by histological analysis (Fig. 7). It is meaningful that *L. sakei* HEM 224 can also affect epithelial barrier health in airways, still confirmation on the protein level for each protein should be provided and bacterial factor(s) are responsible for the reaction need to be clarified.

In this research, we have identified a unique and novel strain that demonstrates beneficial effects on two distinct mucosal sites. What's particularly intriguing is that the observed beneficial effects in each disease model were dependent on the route of administration. In the case of the colitis model, the oral administration of *L. sakei* HEM 224 successfully attenuated colitis by modulating the gut microbiota. However, as mentioned earlier, the oral application of the strain did not ameliorate allergic airway inflammation. Based on these findings, we hypothesize that *L. sakei* HEM 224 may exert its modulatory effects on colitis through a secondary route by stimulating changes in the gut microbiota. Conversely, in the context of airway conditions with allergic inflammation, direct contact between the strain and host components may lead to different regulatory mechanisms. Nevertheless, a comprehensive mechanistic study of *L. sakei* HEM 224 regarding these intriguing responses is still warranted for further applications.

Overall, this study confirmed the safety of *L. sakei* HEM 224 for use as a putative probiotic. Its beneficial functions were evaluated in in vitro and in vivo models. Two inflammatory models targeting the gut and lung were used and *L. sakei* HEM 224 successfully attenuated each inflammatory state with the improvement of epithelial barrier components, including TJPs and mucins. Therefore, *L. sakei* HEM 224 has probiotic potential for use against various mucosal inflammatory disorders.

### Supplementary Information

Supplementary Information.

## Data Availability

WGS sequences were deposed in the database of National Center for Biotechnology Information (NCBI) (https://www.ncbi.nlm.nih.gov/) under the GenBank. The WGS data of *L. sakei* HEM 224 has been deposited under BioProject PRJNA949423 and the accession number for the sequencing data are JARWBH000000000. The main data of this study are available in the article and supplementary information. The raw data are available from the corresponding author on a reasonable request.
