# Author Correction: Multifunctional effects of *Lactobacillus sakei* HEM 224 on the gastrointestinal tract and airway inflammation

**DOI:** 10.1038/s41598-024-68521-5

**Published:** 2024-07-30

**Authors:** Hye‑Shin Kim, Hanna Oh, Bobae Kim, Yosep Ji, Wilhelm H. Holzapfel, Hyeji Kang, Karina Arellano-Ayala

**Affiliations:** 1 Department of Advanced Convergence, Handong Global University, 558, Handong-ro, Pohang, Gyeongbuk 37554 Republic of Korea; 2 HEM Pharma Inc., Pohang, Gyeongbuk 37554 Republic of Korea; 3 Global Green Research and Development Institute, Handong Global University, Pohang, Gyeongbuk 37554 Republic of Korea; 4 Research Institute of Molecular Pathology (IMP), Vienna BioCenter (VBC), Campus-Vienna-Biocenter 1, 1030 Vienna, Austria

Correction to: *Scientific Reports* 10.1038/s41598-023-45043-0, published online 20 October 2023

Karina Arellano-Ayala was omitted from the author list in the original version of this Article.

The Author contributions now reads:

“Hs.K., H.O., B.K., Y.J. W.H. and Hj.K. were involved in the experimental design. Hs.K., H.O. and K.A-A. performed experiments and acquired data. Hs.K., H.O., B.K., W.H. and Hj.K. contribute for the data interpretation, drafting/revision of the manuscript for content. Hs.K mainly wrote the manuscript and prepared figures. All authors reviewed the manuscript.”

The original Article has been corrected.

