# Supplementary material for: Multifunctional effects of Lactobacillus sakei HEM 224 on the gastrointestinal tract and airway inflammation
Source: Sci Rep. 2023 Oct 20;13:17918. doi: 10.1038/s41598-023-45043-0 (PMC10589218; doi:10.1038/s41598-023-45043-0)
Supplement: Supplementary file 1 — Supplementary Information. [file 41598_2023_45043_MOESM1_ESM.docx]

**Supplementary Information**

**Supplementary table 1.** List of primers used for qRT-PCR

**Supplementary table 2.** Hemolysis and biogenic amine production of *L. sakei* HEM 224

**Supplementary table 3.** Minimum inhibitory concentrations of selected antibiotics for *L. sakei* HEM 224

**Supplementary table 4.** Profiles of the detected virulence genes in the genome of *L. sakei* HEM 224

**Supplementary table 5.** SSDP results for *L. sakei* HEM 224

**Supplementary table 6.** Cell adhesion assay results of *L. sakei* HEM 224

**Supplementary table 7.** Fecal SCFA profile in a DSS-induced colitis mouse model

**References**

| **Supplementary table 1. List of primers used for qRT-PCR** | | | |
| --- | --- | --- | --- |
| Target site | Target | Primer sequence (5’-3’) | Reference |
| Colon | *cldn3* | F: CAG ACG TCC GTC AGT TTT CG | [1] |
|  |  | R: CAT GGC TGC TGG ACT TGA AC |  |
|  | *cldn4* | F: CGC TAC TCT TGC CAT TAC G | Primer blast |
|  |  | R: ACT CAG CAC ACC ATG ACT TG |  |
|  | *cldn7* | F: AGG GTC TGC TCT GGT CCT T |  |
|  |  | R: GTA CGC AGC TTT GCT TTC A |  |
|  | *cldn15* | F: CAG CTT CGG TAA ATA TGC CA |  |
|  |  | R: CAG TGG GAC AAG AAA TGG TG |  |
|  | *muc4* | F: GTC TCC CAT CAC GGT TCA GT | [1] |
|  |  | R: TGT CAT TCC ACA CTC CCA GA |  |
|  | *ocldn* | F: AGA CTA CAC GAC AGG TGG GG | [2] |
|  |  | R: CTG CAG ACC TGC ATC AAA AT |  |
|  | *zo1* | F: GCC GCT AAG AGC ACA GCA A | [3] |
|  |  | R: TCC CCA CTC TGA AAA TGA GGA |  |
|  | *tnfa* | F: CAC AAG ATG CTG GGA CAG TGA | Primer blast |
|  |  | R: GAG GCT CCA GTG AAT TCG GA |  |
|  | *il1b* | F: CCT CTC CAG CCA AGC TTC CT | [4] |
|  |  | R: TTT GGA AGC AGC CCT TCA TC |  |
|  | *il6* | F: TCC AGT TGC CTT CTT GGG AC | Primer blast |
|  |  | R: AGT CTC CTC TCC GGA CTT GT |  |
|  | *il17* | F: GGC TGA CCC CTA AGA AAC CC |  |
|  |  | R: AAG CAG TTT GGG ACC CCT TT |  |
|  | *il22* | F: GCT CAG CTC CTG TCA CAT CA |  |
|  |  | R: CAG TTC CCC AAT CGC CTT GA |  |
|  | *il10* | F: GTT GCC AAG CCT TAT CGG AA | [5] |
|  |  | R: CCA GGG AAT TCA AAT GCT CCT |  |
|  | *arbp* | F: TCA CTG TGC CAG CTC AGA AC | Primer blast |
|  |  | R: AAT TTC AAT GGT GCC TCT GG |  |
| Lung | *ccl22* | F: AGG TCC CTA TGG TGC CAA TGT | [6] |
|  |  | R: CGG CAG GAT TTT GAG GTC CA |  |
|  | *ccl24* | F: ATT CTG TGA CCA TCC CCT CAT |  |
|  |  | R: TGT ATG TGC CTC TGA ACC CAC |  |
|  | *cldn18* | F: GAC CGT TCA GAC CAG GTA CA | [7] |
|  |  | R: GCG ATG CAC ATC ATC ACT C |  |
|  | *il13* | F: GCA ACA TCA CAC AAG ACC AGA | [8] |
|  |  | R: GTC AGG GAA TCC AGG GCT AC |  |
|  | *ocldn* | F: CCT CCA CCC CCA TCT GAC TA | [9] |
|  |  | R: CTT CAG GCA CCA GAG GTG TT |  |
|  | *muc5ac* | F: TCC CTT ACC TAA CCA GCA GAA | [10] |
|  |  | R: GGG AGT ACA TGG AGA TGC TGT |  |
|  | *β-actin* | F: GGC TGT ATT CCC CTC CAT CG | [8] |
|  |  | R: CCA GTT GGT AAC AAA TGC CAT GT |  |
| * The primers for ZO-1 were shared in both *in vivo* models. | | | |

| **Supplementary table 2. Hemolysis and biogenic amine production of *L. sakei* HEM 224** | | | | | |
| --- | --- | --- | --- | --- | --- |
| **Strain** | **Hemolysin** | **His** | **Tyr** | **Put** | **Cad** |
| *L. sakei* HEM 224 | ɣ | - | - | - | - |
| *L. rhamnosus* GG | n.a | - | - | - | - |
| *E. coli* ATCC 25922 | n.a | + | + | + | + |
| *B. cereus* ATCC 27348 | ß | n.a | n.a | n.a | n.a |
| The authentic strains *L. rhamnosus* GG, *E. coli* ATCC 25922 and *B. cereus* ATCC 27348 served as references. His: histamine, Tyr: tyramine, Put: putrescin, Cad: cadaverine, α: alpha, ß: beta, ɣ: gamma, n.a: not analyzed, - : no significant color change of bromocresol purple in BA production detection agar. | | | | | |

| **Supplementary table 3. Minimum inhibitory concentrations of *L. sakei* HEM 224** | | | | | | | | |
| --- | --- | --- | --- | --- | --- | --- | --- | --- |
| **Strain** | **Minimum inhibitory concentration (mg/L)** | | | | | | | |
|  | **AMP** | **ERY** | **GEN** | **TET** | **STR** | **CLI** | **KAN** | **CHL** |
| *L. sakei* HEM 224 | 0.5 (S) | ≤0.25 (S) | 4 (S) | 4 (S) | 32 (S) | ≤0.25 (S) | 16 (S) | 4 (S) |
| EFSA Cut-off* | 4 | 1 | 16 | 8 | 64 | 4 | 64 | 4 |
| AMP: ampicillin; CHL: Chloramphenicol; CLI: Clindamycin; ERY: Erythromycin; GEN: Gentamicin; KAN: Kanamycin; STR: Streptomycin; TET: Tetracycline; n.r.: not required. *Cut-off values stablished by the European Committee on Antimicrobial Susceptibility Testing (EUCAST, http:// www.eucast.org/), EFSA. | | | | | | | | |

**Supplementary table 4. Profiles of the detected virulence genes in the genome of *L. sakei* HEM 224**

| **Antimicrobial** | **Class** | **WGS-predicted phenotype** | **Antimicrobial** | **Class** | **WGS-predicted phenotype** |
| --- | --- | --- | --- | --- | --- |
| formaldehyde | aldehydes | No resistance | unknown fluoroquinolone | fluoroquinolone | No resistance |
| spectinomycin | aminocyclitol | No resistance | ciprofloxacin | fluoroquinolone | No resistance |
| tobramycin | aminoglycoside | No resistance | nalidixic acid | fluoroquinolone | No resistance |
| hygromycin | aminoglycoside | No resistance | sulfamethoxazole | folate pathway antagonist | No resistance |
| isepamicin | aminoglycoside | No resistance | trimethoprim | folate pathway antagonist | No resistance |
| butirosin | aminoglycoside | No resistance | fosfomycin | fosfomycin | No resistance |
| astromicin | aminoglycoside | No resistance | vancomycin | glycopeptide | No resistance |
| lividomycin | aminoglycoside | No resistance | teicoplanin | glycopeptide | No resistance |
| butiromycin | aminoglycoside | No resistance | temperature | heat | No resistance |
| paromomycin | aminoglycoside | No resistance | lincomycin | lincosamide | No resistance |
| gentamicin | aminoglycoside | No resistance | clindamycin | lincosamide | No resistance |
| ribostamycin | aminoglycoside | No resistance | oleandomycin | macrolide | No resistance |
| kanamycin | aminoglycoside | No resistance | unknown macrolide | macrolide | No resistance |
| amikacin | aminoglycoside | No resistance | erythromycin | macrolide | No resistance |
| neomycin | aminoglycoside | No resistance | telithromycin | macrolide | No resistance |
| sisomicin | aminoglycoside | No resistance | tylosin | macrolide | No resistance |
| unknown aminoglycoside | aminoglycoside | No resistance | carbomycin | macrolide | No resistance |
| arbekacin | aminoglycoside | No resistance | azithromycin | macrolide | No resistance |
| fortimicin | aminoglycoside | No resistance | spiramycin | macrolide | No resistance |
| kasugamycin | aminoglycoside | No resistance | metronidazole | nitroimidazole | No resistance |
| apramycin | aminoglycoside | No resistance | linezolid | oxazolidinone | No resistance |
| dibekacin | aminoglycoside | No resistance | hydrogen peroxide | peroxides | No resistance |
| netilmicin | aminoglycoside | No resistance | florfenicol | phenicol | No resistance |
| streptomycin | aminoglycoside | No resistance | unknown phenicol | phenicol | No resistance |
| ampicillin | beta-lactam | No resistance | chloramphenicol | phenicol | No resistance |
| temocillin | beta-lactam | No resistance | tiamulin | pleuromutilin | No resistance |
| cephalothin | beta-lactam | No resistance | colistin | polymyxin | No resistance |
| piperacillin+clavulanic acid | beta-lactam | No resistance | mupirocin | pseudomonic acid | No resistance |
| amoxicillin+clavulanic acid | beta-lactam | No resistance | cetylpyridinium chloride | quaternary ammonium compounds | No resistance |
| piperacillin+tazobactam | beta-lactam | No resistance | benzylkonium chloride | quaternary ammonium compounds | No resistance |
| ticarcillin+clavulanic acid | beta-lactam | No resistance | chlorhexidine | quaternary ammonium compounds | No resistance |
| cephalotin | beta-lactam | No resistance | rifampicin | rifamycin | No resistance |
| cefoxitin | beta-lactam | No resistance | unknown rifamycin | rifamycin | No resistance |
| penicillin | beta-lactam | No resistance | fusidic acid | steroid antibacterial | No resistance |
| piperacillin | beta-lactam | No resistance | virginiamycin m | streptogramin a | No resistance |
| amoxicillin | beta-lactam | No resistance | dalfopristin | streptogramin a | No resistance |
| meropenem | beta-lactam | No resistance | quinupristin+dalfopristin | streptogramin a | No resistance |
| cefepime | beta-lactam | No resistance | pristinamycin iia | streptogramin a | No resistance |
| imipenem | beta-lactam | No resistance | virginiamycin s | streptogramin b | No resistance |
| cefixime | beta-lactam | No resistance | pristinamycin ia | streptogramin b | No resistance |
| cefotaxime+clavulanic acid | beta-lactam | No resistance | quinupristin | streptogramin b | No resistance |
| ceftriaxone | beta-lactam | No resistance | tigecycline | tetracycline | No resistance |
| ceftazidime | beta-lactam | No resistance | doxycycline | tetracycline | No resistance |
| ticarcillin | beta-lactam | No resistance | unknown tetracycline | tetracycline | No resistance |
| ampicillin+clavulanic acid | beta-lactam | No resistance | tetracycline | tetracycline | No resistance |
| cefotaxime | beta-lactam | No resistance | minocycline | tetracycline | No resistance |
| ceftazidime+avibactam | beta-lactam | No resistance | fluoroquinolone | under_development | No resistance |
| ertapenem | beta-lactam | No resistance |  |  |  |
| aztreonam | beta-lactam | No resistance |  |  |  |
| unknown beta-lactam | beta-lactam | No resistance |  |  |  |

| **Supplementary table 5. Simulated stomach duodenum passage (SSDP) results of *L. sakei* HEM 224 compared with the control, *L. rhamnosus* GG** | | | | | |
| --- | --- | --- | --- | --- | --- |
| **Strain** | **Initial** | **After stomach pass** | | **After duodenum pass** | |
|  | **Log CFU/mL** | **Log CFU/mL** | **%** | **Log CFU/mL** | **%** |
| *L. sakei* HEM 224 | 8.50 ± 0.13 | 6.50 ± 0.07 | 1.01 | 5.76 ± 0.07 | 0.18 |
| *L. rhamnosus* GG | 7.90 ± 0.24 | 5.09 ± 0.21 | 0.21 | 4.41 ± 0.72 | 0.05 |
|  | | | | | |

| **Supplementary table 6. Cell adhesion assay results of *L. sakei* HEM 224 and the control, *L. rhamnosus* GG** | |
| --- | --- |
| **Strain** | **Relative percent adhesion (%)** |
| *L. sakei* HEM 224 | 3.60±1.18 |
| *L. rhamnosus* GG | 3.67±0.01 |

**Supplementary table 7. Fecal SCFA profile in a DSS-induced colitis mouse model**

| **Parameters** | **Group** | | |
| --- | --- | --- | --- |
|  | **CTRL** | **DSS + PBS** | **DSS + LS224** |
| Acetate (%) | 74.67 | 85.13 | 72.98 |
| Propionate (%) | 19.90 | 13.96 | 9.40 |
| Butyrate (%) | 5.43 | 0.91 | 17.62 |
| Butyrate / Acetate | 0.07 | 0.01 | 0.24 |
| Butyrate / Propionate | 0.27 | 0.07 | 1.87 |

**References**

1. Kim, W. K. *et al*. Administration of *Lactobacillus fermentum* KBL375 Causes Taxonomic and Functional Changes in Gut Microbiota Leading to Improvement of Atopic Dermatitis. *Front. Mol. Biosci.* **6**, 92 <https://doi.org/10.3389/fmolb.2019.00092> (2019).
2. Chelakkot, C. *et al*. Intestinal epithelial cell-specific deletion of PLD2 alleviates DSS-induced colitis by regulating occludin. *Sci. Rep.* **7**(1), 1573 <https://doi.org/10.1038/s41598-017-01797-y> (2017).
3. Chiaro, T. R. *et al.* A member of the gut mycobiota modulates host purine metabolism exacerbating colitis in mice. *Sci. Transl. Med.* **9**(380), eaaf9044 <https://doi.org/10.1126/scitranslmed.aaf9044> (2017).
4. Flück, K., Breves, G., Fandrey, J., & Winning, S. Hypoxia-inducible factor 1 in dendritic cells is crucial for the activation of protective regulatory T cells in murine colitis. *Mucosal Immunol*. **9**(2), 379–390 <https://doi.org/10.1038/mi.2015.67> (2016).
5. Morampudi, V. *et al*. The goblet cell-derived mediator RELM-β drives spontaneous colitis in Muc2-deficient mice by promoting commensal microbial dysbiosis. *Mucosal Immunol*. **9**(5), 1218–1233 <https://doi.org/10.1038/mi.2015.140>. (2016).
6. Yi, S. *et al*. Eosinophil recruitment is dynamically regulated by interplay among lung dendritic cell subsets after allergen challenge. *Nat. Commun.* **9**(1), 3879 <https://doi.org/10.1038/s41467-018-06316-9> (2018).
7. Yang, R., Tan, M., Xu, J., & Zhao, X. Investigating the regulatory role of ORMDL3 in airway barrier dysfunction using *in vivo* and *in vitro* models. *Int. J. Mol. Med.* **44**(2), 535–548 <https://doi.org/10.3892/ijmm.2019.4233> (2019).
8. Shibata, S. *et al*. Basophils trigger emphysema development in a murine model of COPD through IL-4-mediated generation of MMP-12-producing macrophages. *Proc. Natl. Acad. Sci. U.S.A.* **115**(51), 13057–13062 <https://doi.org/10.1073/pnas.1813927115> (2018).
9. Klaßen, C. *et al*. Airway Epithelial Cells Are Crucial Targets of Glucocorticoids in a Mouse Model of Allergic Asthma. *J. Immunol.*, **199**(1), 48–61 <https://doi.org/10.4049/jimmunol.1601691> (2017).
10. Tetaert, D. *et al*. Dietary n-3 fatty acids have suppressive effects on mucin upregulation in mice infected with *Pseudomonas aeruginosa*. *Respir. Res.*, **8**(1), 39 <https://doi.org/10.1186/1465-9921-8-39> (2007).
